# Integrin α6-Targeted Molecular Imaging of Central Nervous System Leukemia in Mice

**DOI:** 10.3389/fbioe.2022.812277

**Published:** 2022-02-23

**Authors:** Wenbiao Zhang, Yongjiang Li, Guanjun Chen, Xiaochun Yang, Junfeng Hu, Xiaofei Zhang, Guokai Feng, Hua Wang

**Affiliations:** ^1^ Department of Medical Imaging, Sun Yat-sen University Cancer Center, Guangzhou, China; ^2^ State Key Laboratory of Oncology in South China, Collaborative Innovation Center for Cancer Medicine, Sun Yat-sen University Cancer Center, Guangzhou, China; ^3^ Department of Nuclear Medicine, Sun Yat-sen University Cancer Center, Guangzhou, China; ^4^ Department of Hematological Oncology, Sun Yat-sen University Cancer Center, Guangzhou, China

**Keywords:** leukemia, molecular imaging, integrin α6, positron emission tomography, central nervous system leukemia

## Abstract

Central nervous system leukemia (CNS-L) is caused by leukemic cells infiltrating into the meninges or brain parenchyma and remains the main reason for disease relapse. Currently, it is hard to detect CNS-L accurately by clinically available imaging models due to the relatively low amount of tumor cells, confined blood supply, and the inferior glucose metabolism intensity. Recently, integrin α6-laminin interactions have been identified to mediate CNS-L, which suggests that integrin α6 may be a promising molecular imaging target for the detection of CNS-L. The acute lymphoblastic leukemia (ALL) cell line NALM6 stabled and transfected with luciferase was used to establish the CNS-L mouse model. CNS-L-bearing mice were monitored and confirmed by bioluminescence imaging. Three of our previously developed integrin α6-targeted peptide-based molecular imaging agents, Cy5-S5 for near-infrared fluorescence (NIRF), Gd-S5 for magnetic resonance (MR), and ^18^F-S5 for positron emission tomography (PET) imaging, were employed for the molecular imaging of these CNS-L-bearing mice. Bioluminescence imaging showed a local intensive signal in the heads among CNS-L-bearing mice; meanwhile, Cy5-S5/NIRF imaging produced intensive fluorescence intensity in the same head regions. Moreover, Gd-S5/MR imaging generated superior MR signal enhancement at the site of meninges, which were located between the skull bone and brain parenchyma. Comparatively, MR imaging with the clinically available MR enhancer Gd-DTPA did not produce the distinguishable MR signal in the same head regions. Additionally, ^18^F-S5/PET imaging also generated focal radio-concentration at the same head regions, which generated nearly 5-times tumor-to-background ratio compared to the clinically available PET radiotracer ^18^F-FDG. Finally, pathological examination identified layer-displayed leukemic cells in the superficial part of the brain parenchyma tissue, and immunohistochemical staining confirmed the overexpression of the integrin α6 within the lesion. These findings suggest the potential application of these integrin α6-targeted molecular imaging agents for the accurate detection of CNS-L.

**GRAPHICAL ABSTRACT:**
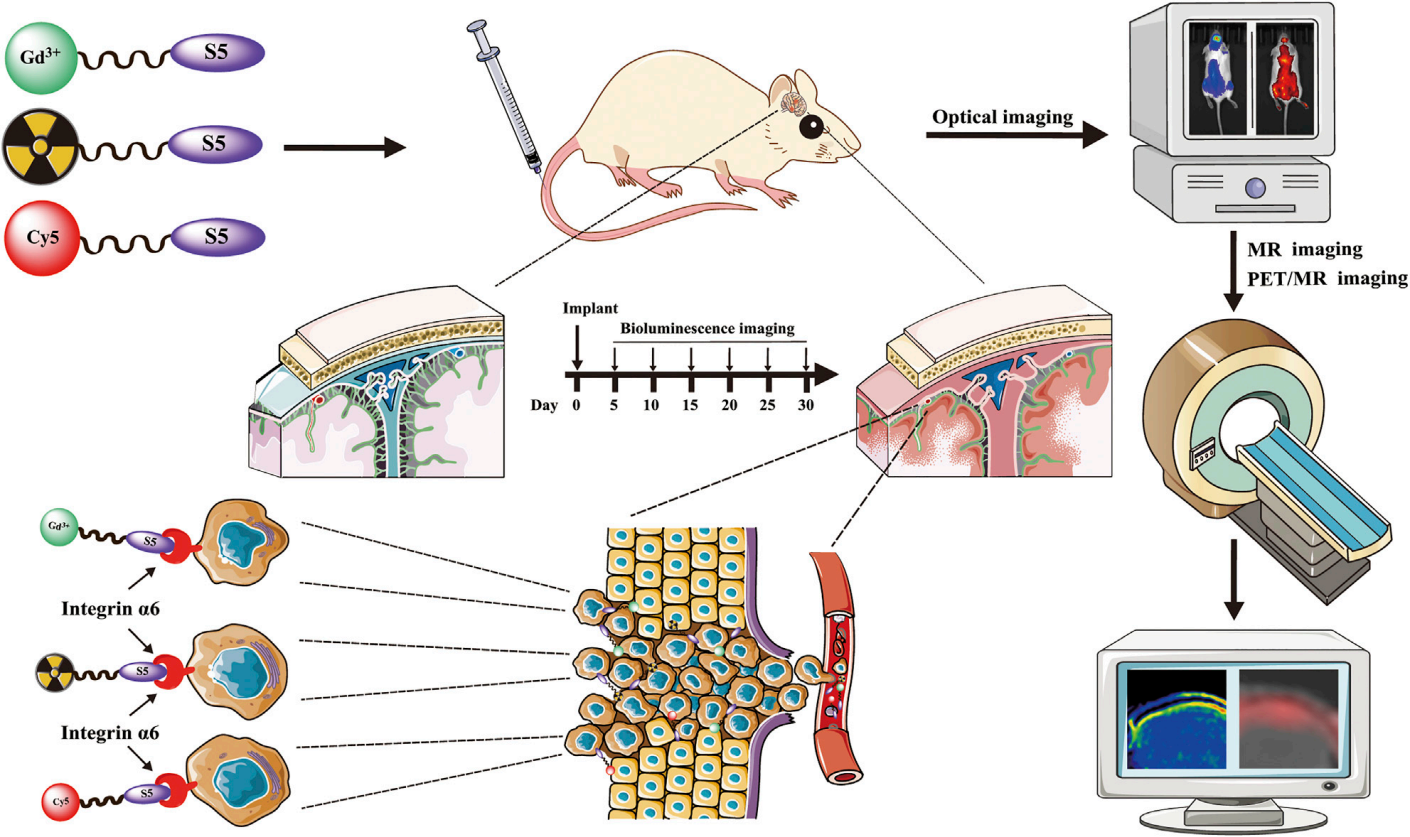


## Introduction

Acute lymphoblastic leukemia (ALL) is a malignant proliferation of lymphocytes that could invade the bone marrow, blood, and extramedullary tissues or organs, with an estimated incidence of about 157/100,000 and a distinctive prevalence in children, especially in those at 1–4 years old (Siegel et al., 2019; Malard and Mohty, 2020). About 80–90% patients could achieve a complete response after multiagent chemotherapy. However, central nervous system involvement of ALL, caused by leukemic cells infiltrating into the meninges or brain parenchyma, which is also called central nervous system leukemia (CNS-L), remains the main reason for disease relapse during the remission period. Because most chemotherapeutic drugs could not penetrate the blood–brain barrier, leukemic cells hidden in the central nervous system could not be effectively eliminated and become the origin of extramedullary leukemia recurrence (Pinnix et al., 2018; Zhang C et al., 2020). Nevertheless, accurate diagnosis of the central nervous system (CNS) involvement is a major clinical challenge that commonly leads to delayed or excessive treatment (Frishman-Levy and Izraeli, 2017; Jin et al., 2018). Due to the diagnosis insufficiency, prophylactic intrathecal chemotherapy (methotrexate in most regimens) is routinely administered for all patients in the clinical setting regardless of their CNS status, and chemotherapy-induced CNS toxicity always accompanied numerous adverse clinical manifestations including impaired consciousness, focal deficits, seizures, and headaches (Vagace et al., 2012). In addition, patients evaluated at a high risk of CNS infiltration routinely received additional cranial irradiation, which could further exacerbate neurocognitive impairment and the risk of secondary malignancies (Zhou et al., 2020).

The current gold standard for assessing CNS-L is pathological detection of leukemic cells in the cerebrospinal fluid through lumbar puncture (Bürger et al., 2003). However, the examination method has a relatively high false-negative rate, and the positivity is hysteretic than the primary CNS involvement, which highlights the urgent need for novel diagnostic methods. New strategies aimed at improving the diagnostic sensitivity of cerebrospinal fluid examination by qPCR or flow cytometry encountered methodological challenges (Yousafzai et al., 2019; Thastrup et al., 2020). Imaging diagnosis by detecting CNS lesions could be more district and intuitive; however, conventional imaging examinations including enhanced computerized tomography (CT), magnetic resonance imaging (MRI), or ^18^F-FDG positron emission tomography (PET) showed limited ability in detecting CNS-L lesions because of a relatively low amount of tumor cells, confined blood supply, and inferior glucose metabolism intensity compared with other solid tumor lesions (Ranta et al., 2017a; Ranta et al., 2017b). Based on the premises, the development of a specific molecular probe for imaging could provide a novel way for the targeted imaging of CNS-L.

Integrins are heterodimeric transmembrane receptors consisting of α and β subunits, each with a single-pass transmembrane domain and a typically short cytoplasmic domain (Erdreich-Epstein et al., 2005; Zhu et al., 2007). Integrin α6 is encoded by the ITGA6 gene and dimerizes with integrin β1 or β4 to form integrin α6β1 and α6β4 (Takada et al., 2007). Integrin α6 normally expresses on the cell surface and acts as the mediator of intracellular and extracellular matrix adhesion (Krebsbach and Villa-Diaz, 2017). Numerous studies have revealed that integrin α6 was highly expressed in tumor cells of ALL patients and the corresponding cell lines (Blase et al., 1996). According to the GEPIA database, ITGA6 was significantly overexpressed in leukemia cells than the paired normal blood cells and the brain tissues (http://gepia.cancer-pku.cn/). Recent studies have found that ALL chemoresistance was dependent on ALL cells adhering to the stroma through the adhesion of integrin α6, and its expression mediated the invasion to the CNS tissues *via* neuromigration pathways (Yao et al., 2018; Gang et al., 2020; Lenk et al., 2020). Thus, integrin α6 could serve as a potential molecular target for the imaging of CNS-L lesions.

In our previous study, we have identified a peptide CRWYDENAC (dubbed RWY) that could bind specifically to integrin α6 by phage display technology (Feng G et al., 2019). We further translated the RWY peptide into an integrin α6-targeted PET probe and MRI probe that have been successfully applied for tumor imaging of the integrin α6 expression (Feng GK et al., 2019; Xiao et al., 2019; Zhang Y et al., 2020; Gao et al., 2020). Recently, our group improved the characteristics of the peptide by alanine scanning and obtained a novel integrin α6-targeting peptide CRWYDANAC (dubbed S5) which exhibited a higher specificity and affinity to integrin α6 (Mei et al., 2020; Lin et al., 2021). On these bases, we further developed the targeted molecular imaging probes for near-infrared fluorescence (NIRF) imaging, MRI, and PET based on the S5 peptide, which is called Cy5-S5, Gd-S5, and ^18^F-S5 in short. In this study, the affinity of the synthesized S5 peptide to ALL cell lines was verified, and the imaging of CNS-L mouse models using NIRF, MRI, and PET scanners was conducted to test the imaging efficacy of the targeted imaging probes for CNS-L.

## Materials and Methods

### Cells and Animals

The human ALL cell lines including NALM6, Reh, Jurkat, and CCRF-CEM were purchased from the American Type Culture Collection (ATCC) and cultured in RPMI 1640 medium supplemented with 10% fetal bovine serum and 0.5% penicillin–streptomycin and incubated at 37 °C/5%CO_2_. To facilitate tumor monitoring, luminescent cells NALM6-luciferase were generated by stable transfection with luciferase. Female NOD/SCID mice (6 weeks old) were purchased from Vita River, Charles River Laboratories, China (Beijing, China). NOD/SCID mice were injected *via* the tail vein with 1 × 10^6^ luciferased cells suspended in 150 µl of sterile phosphate-buffered saline (PBS). Bioluminescence imaging was applied to monitor the leukemic burden. All animal experiments were approved by the Institutional Animal Care and Use Committee (IACUC) at the Sun Yat-sen University Cancer Center (IACUC approval number L102012020070M), and the welfare and treatment of the laboratory animals were in accordance with the corresponding animal management regulations.

### Preparation of S5 Peptide-Based Imaging Probes for NIRF, MR, and PET

The integrin α6-targeted peptide S5 was synthesized by the standard Fmoc-based solid-phase synthesis and provided by Chinese Peptide Company (Hangzhou, China). In order to make a better combination of S5 and Gd and to reduce the complexity of the process, we used the S5 peptide sequence as a straight peptide in the synthesis of the peptide Gd-S5, while the other two synthetic peptides were cyclic peptides. Briefly, DOTA-S5 or NOTA-S5 was synthesized through a condensation reaction between carboxyl groups in DOTA or NOTA and amine groups in reverse S5. The crude product was eluted with a gradient of acetonitrile, and the end product was purified by high-performance liquid chromatography (HPLC), followed by characterization using matrix-assisted laser desorption/ionization time-of-flight (MALDI-TOF) mass spectrometry (Bruker Daltonics, Germany). Then, Cy5-S5 was compounded by conjugating the Cy5 to the S5 peptide through an amidation reaction, and Gd-S5 was synthesized by complexation of Gd with the DOTA-S5.

As ^18^F has a relatively fast rate of decay, ^18^F-S5 would be synthesized each time before use by radiolabeling the NOTA-S5 with ^18^F. In brief, the S5 peptide solution was freeze-dried with the lyophilized kits, with an 8 nmol S5 peptide and 6 nmol AlCI_3_.6H2O in each vial unit. The reaction procedure was performed as follows: 1 vial unit was added with 65 μl of ^18^F (approximately 10 mCi) in DI water, 5 μl of acetic acid, and 330 μl ethanol and heated for 10 min at 100°C. The reaction mixture was diluted with 10 ml of water and trapped in a Varian Bond Elut C18 column (100 mg). The column was washed with 10 ml of water and then eluted with 400 μl of ethanol twice. Then, the eluate was air-dried by nitrogen. Finally, the end product was dissolved with PBS before injection.

### Flow Cytometry

NALM6, Ref, Jurkat, and CCRF-CEM cells were seeded into six-well plates, respectively, and then incubated with 10 µl/10^6^ cell concentration of the anti-integrin α6 antibody (R&D, FAB13501P) and IgG2A (R&D, IC006P) at 4°C for 30 min, protected from light. After incubating for 30 min, the cells were washed with PBS three times and resuspended in 500 μl of PBS. The fluorescence intensity was measured by flow cytometry (Beckman Coulter, CytoFLEX S, United States).

### Immunofluorescence

Approximately 1 × 10^6^ NALM6 cells were seeded on cover slips in 24-well plates and incubated for 24 h. Subsequently, the 80 μM S5 peptide was added into the culture medium and incubated with cells for 4 h, while the blank control group was added to the same volume of RPMI 1640 medium. After that, cells of each group were washed with PBS five times, fixed with 4% paraformaldehyde for 15 min, permeabilized with 0.1% Triton X-100 (Sigma-Aldrich, Germany), and blocked with 1% BSA for 30 min. The cover slips were incubated with the anti-integrin α6 antibody (Abcam, ab181551) at 4°C overnight, followed by incubation with streptavidin-Cy3 (Thermo Fisher 434315, United States) and the goat anti-mouse Alexa Fluor 488 secondary antibody (Abcam, ab150113) for 1 h at room temperature in a dark chamber. Finally, cover slips were incubated with 1 μg/ml DAPI and mounted with slides with ProLong gold antifade (Invitrogen P26930, United States). Fluorescence images were visualized and captured under a confocal microscopy confocal laser-scanning system (Zeiss, LSM980, Germany) at 40× and 100× magnification. Co-localization was analyzed by ImageJ (http://rsbweb.nih.gov/ij/) and the co-localization finder plug-in.

### NIRF Imaging

Both bioluminescence and NIRF imaging are performed using IVIS Spectrum equipment manufactured by PerkinElmer, United States. We performed bioluminescence imaging on days 5, 10, 15, 20, 25, and 30 after the tail vein injection of leukemic cells until the imaging results confirmed the presence of focal luminescence intensity around the head of the mice. Bioluminescence imaging was performed starting 5 min after the intraperitoneal injection of luciferin. NIRF was acquired with an excitation at 640 nm and emission at 680 nm (exposure time: 3 s). Mice with CNS-L confirmed by bioluminescence were used for NIRF imaging studies on the following day. Mice were anesthetized with 3% isoflurane and maintained under anesthesia with 1–2% isoflurane and oxygen. An injection of Cy5-S5 and Cy5-CG7C solutions was freshly prepared in saline, and the solution was stabilized for 20 min before injection. Each mouse was injected intravenously with an equivalent of 10 nmol (0.5 mg/Kg) of Cy5-S5 and Cy5-CG7C. Fluorescence signals from the tumor were recorded before and 48 h after intravenous injection of the probes.

### MR Imaging

Bioluminescence confirmed CNS-L mice were used for MR and PET imaging studies in the next few days after the NIRF imaging. MR imaging was performed using a Philips Achieva 3.0 T system. Mice were anesthetized with 2,2,2-Tribromoethanol (100 μl/g), fixed on the holder, and placed into the mouse-imaging coil. Gd-S5 or a control agent was delivered by the tail vein at a dose of 0.03 mmol of Gd/kg for the T1 mapping sequence in NALM6-luciferase tumor-bearing mice. The clinical commonly used MR contrast medium gadolinium-diethylenetriamine pentaacetic acid (Gd-DTPA), which is a nonspecific agent, was used as control. On two separate days, mice received either a control enhance-agent (0.03 mmol of Gd/kg) or Gd-S5 (0.03 mmol of Gd/kg) injection and subsequent MR imaging, with at least 3 days between the scans to ensure most of the gadolinium were cleared. Axial T1-weighted images were acquired by using a gradient-echo sequence with the following parameters: TR/TE = 8.771/2.878 ms, FOV = 4.0 cm, slice thickness = −1 mm, slice spacing = 0.5, pixel spacing = 0.234 mm × 0.234 mm, and matrix = 128 × 128.

### PET Imaging

PET imaging was performed using a hybrid 3.0T PET/MR scanner (uPMR 790, United Imaging Healthcare). As described above, mice were anesthetized with 2,2,2-Tribromoethanol (100 μl/g), fixed on the holder, and placed into the mouse-imaging coil. Approximately, 3.7 × 10^6^ Bq (100 μCi) of ^18^F-S5 or the clinical commonly used PET tracer ^18^F-fluorodeoxyglucose (FDG) as control was injected *via* the tail vein before PET imaging. PET scans of the tumor-bearing mice were performed 60 min after injection, with the following parameters: TR/TE = 8.771/2.878 ms, FOV = 4.0 cm, slice thickness = 1 mm, slice spacing = 0.5, pixel spacing = 0.234 mm × 0.234 mm, and matrix = 128 × 128.

### HE Staining and Immunohistochemistry

At the end of imaging experiments, the mice were sacrificed by cervical dislocation and immediately dissected. The head part was collected, and haired skin and soft tissue were removed from the cranial surface. Paraffin sections (3 μm) were stained with hematoxylin and eosin (H&E) for routine histologic practice. Immunohistochemical (IHC) staining was performed following the conventional procedure, as we reported previously (Feng GK et al., 2019). Briefly, paraffin sections were dewaxed into xylene, rehydrated through graded alcohol, and microwaved for antigen retrieval. Blocking to inhibit the endogenous peroxidase activity and nonspecific binding, the sections were incubated with an anti-integrin α6 antibody (Abcam, ab181551, and 1:150) overnight at 4°C, followed by an HRP-conjugated polyclonal secondary antibody (1:200) at room temperature for 1 h. Finally, the positive immunoreactivity was visualized by staining with DAB (Zhongshan Jinqiao, ZLI-9017, China) and observed under a microscope (Nikon Eclipse, Japan).

### Statistical Analysis

Statistical analysis was performed with SPSS 19.0 software (SPSS Inc., Chicago, United States). The significance was tested by the two-tailed Student’s t-test, and a *p* value less than 0.05 was considered to be statistically significant.

## Results

### Surface Expression of Integrin α6 in Human ALL Cell Lines

**FIGURE 1:**
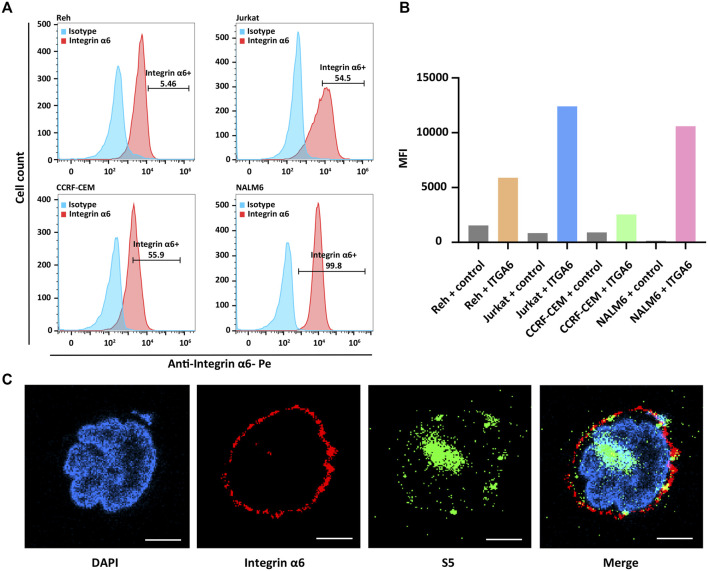
Surface expression of integrin α6 in human ALL cell lines and binding of the S5 peptide to NALM6 cells. **(A)** Flow cytometry identified the surface expression of integrin α6 in human ALL cell lines including NALM6, Ref, Jurkat, and CCRF-CEM, and **(B)** the highest expression of integrin α6 was observed in NALM6 cells. **(C)** Confocal microscopy confirmed the cellular localization of integrin α6 and the S5 peptide in NALM6 cells (Scale bar, 4 μm).

The surface expression of integrin α6 was assayed by flow cytometry in human ALL cell lines including NALM6, Ref, Jurkat, and CCRF-CEM. As expected, flow cytometry confirmed the surface expression of integrin α6 in all the cell lines (Figure 1A). In Figure 1B, we describe the mean fluorescence intensity (MFI) of different cell flow assays. MFI means total fluorescence intensity divided by the number of positive cells. The highest expression of integrin α6 was observed in NALM6 cells with a positive rate of 99.8% (Figure 1B), which was used to establish ALL models for subsequent imaging experiments.

### Synthesis and Characterization

The S5 peptide was synthesized using Fmoc-based solid-phase synthesis. Then, the S5 peptide was condensed with a PEG4 spacer and the Cy5, NOTA, or DOTA structure to furnish the Cy5-S5, NOTA-S5, and DOTA-S5. The purity of Cy5-S5, NOTA-S5, and DOTA-S5 was over 95% by high-performance liquid chromatography (HPLC). The molecular weight was measured to be 1985, 1,632, and 1,435 by MALDI-TOF mass spectrometry, which was in agreement with the theoretical molecular weight calculated from the predicted amino acid sequence. Then, the DOTA-S5 was complexed with Gd, and the NOTA-S5 peptide was radiolabeled with the radionuclide ^18^F to form the MR enhancer Gd-S5 and the PET radiotracer ^18^F-S5 (Supplementary Figure S1). The structural formula used for the Gd-S5 peptide used for MR imaging is a straight peptide, based on the convenience and requirements of the synthesis process and the fact that our preliminary experiments have confirmed that the binding of cyclic and straight peptides is equivalent. The corresponding results are supplemented in Supplementary Figures S2A, S2B. In addition, the binding experiments with S5 carrying NOTA groups found that the attachment of NOTA did not affect the binding affinity of the peptide (Supplementary Figures S2C, S2D).

### Biding of the S5 Peptide to NALM6 Cells and NIRF Imaging

**FIGURE 2:**
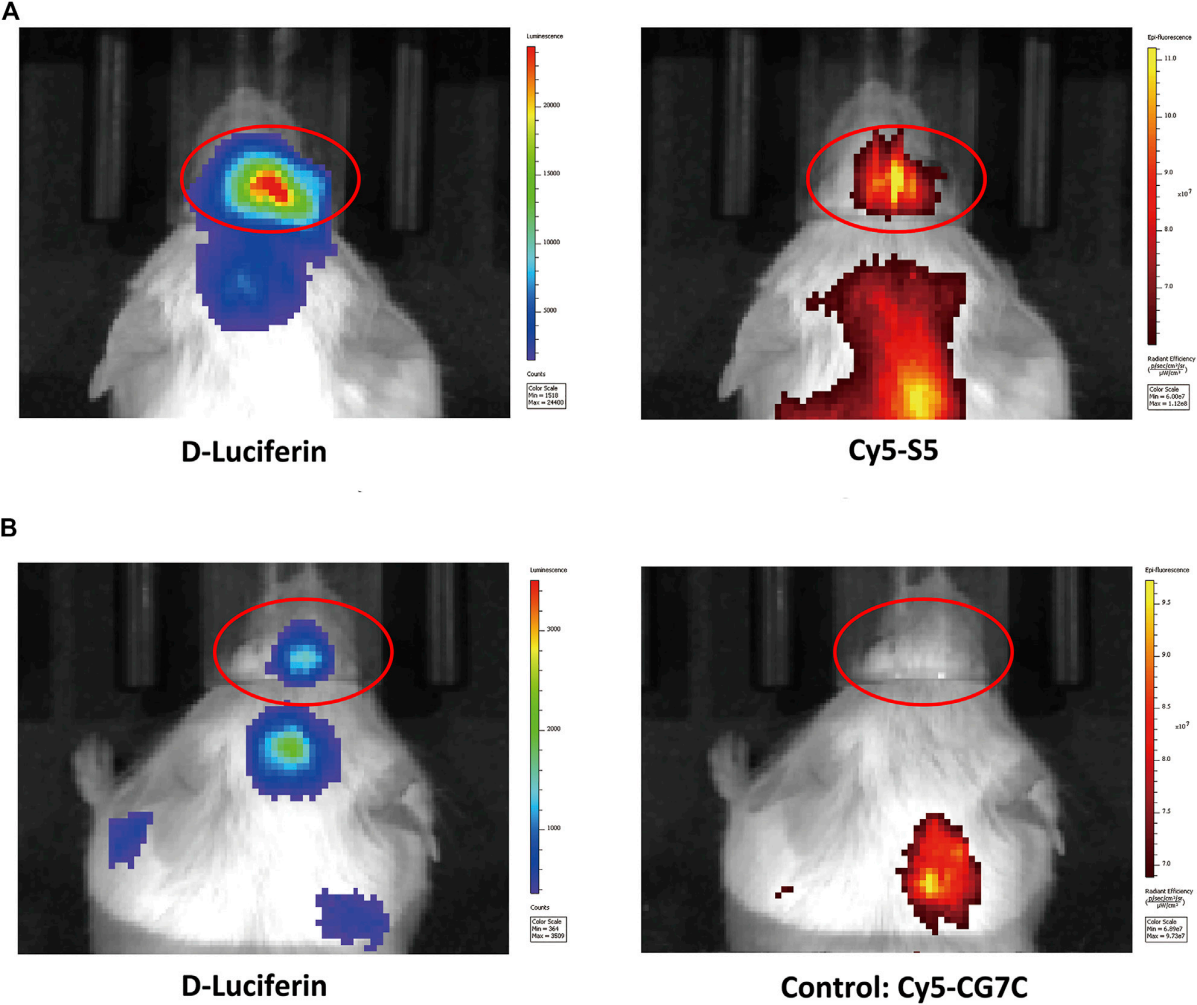
NIFR imaging with Cy5-S5 in ALL mouse models verified by bioluminescence imaging. **(A)** Cy5-S5 exhibited intensive fluorescence intensity in the similar tumor location of bioluminescence imaging, while **(B)** Cy5-CG7C did not show significant fluorescence intensity.

The cellular localization of the S5 peptide in NALM6 cells was visualized by confocal microscopy. Green fluorescence represented the S5 polypeptide, which entered the cytoplasm through cytocytosis after binding to the cell membrane. Red fluorescence represented the integrin α6 antibody, which was bound to the cell membrane and was then immobilized on the cell membrane. According to the fluorescence microscopy data, the S5 peptide was enriched and localized at the nuclei of NALM6 cells after 4 h of co-culturing (Figure 1C), indicating the targeted ability of the S5 peptide to integrin α6 that was dominantly distributed on the nuclei. To further investigate the *in vivo* tumor-binding ability of the S5 peptide, NIRF imaging with Cy5-S5 and Cy5-CG7C (negative control) was conducted. The NALM6-luciferase tumor-bearing mice were verified by bioluminescence imaging with a focal luminescence intensity around the head, and in NIRF imaging, Cy5-S5 exhibited intensive fluorescence intensity in the similar tumor location (Figure 2A), while the control imaging group with Cy5-CG7C did not show significant fluorescence intensity (Figure 2B).

### MR Imaging in NALM6-Luciferase Tumor-Bearing Mice

**FIGURE 3:**
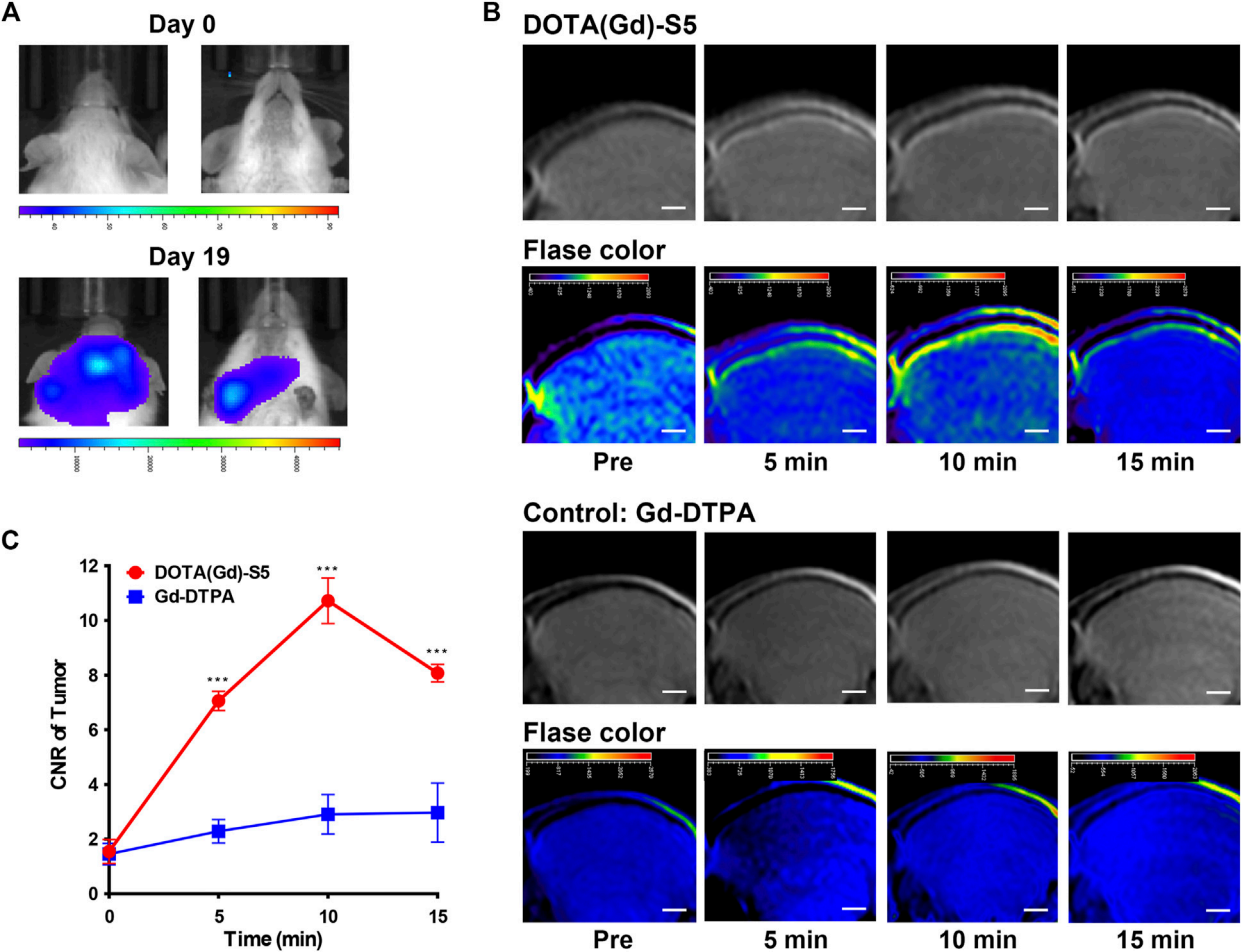
MR imaging with Gd-S5 in ALL mouse models. **(A)** Bioluminescence imaging exhibited focal luminescence intensity around the head 19 days after injection of NALM6-luciferased cells. **(B)** T1-weighted MR images at the baseline and 5, 10, and 15 min post-injection of Gd-S5 and the control probe Gd-DTPA (Scale bar, 1 mm). **(C)** Gd-S5 generated a significantly higher signal enhancement at the site of meninges which were located between the skull bone and brain parenchyma at 5, 10, and 15 min post-injection, with the greatest gap at about 10 min post-injection (*n* = 3).

Similarly, a leukemia CNS-L mouse model using NALM6 cells with the luciferase expression by tail vein injection was established. Approximately 3 weeks after tumor cell injection, the presence of focal luminescence intensity around the head was confirmed by bioluminescence imaging (Figure 3A). T1-weighted MR imaging was conducted before and at different time points after Gd-S5 or Gd-DTPA injection. To improve visibility, MR signals were also displayed in rainbow pseudo color. As shown in Figure 3B, Gd-S5 gradually produced contrast enhancement signals at the site of meninges located between the skull bone and brain parenchyma at 5 min post-injection, which conformed to the primary intracranial infiltrating location of CNS-L (Yao et al., 2018), reaching the peak at 10 min, and gradually decreased thereafter. The control group with Gd-DTPA injection did not show obvious enhancement signals. The signal intensity comparison according to the post-injection duration is presented in Figure 3C, indicating that Gd-S5 generated significantly higher signals than Gd-DTPA.

### PET Imaging in NALM6-Luciferase Tumor-Bearing Mice

**FIGURE 4:**
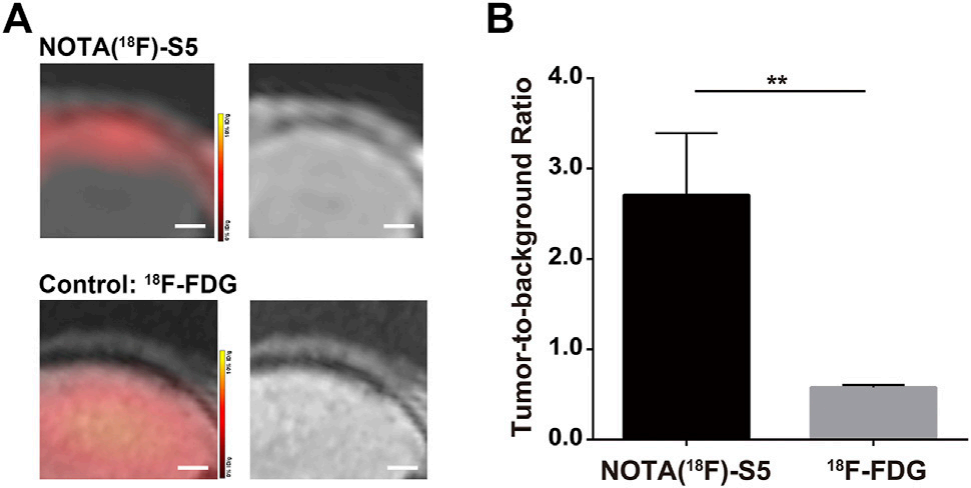
^18^F-S5 PET/MR imaging in ALL mouse models. **(A)** ^18^F-S5 generated focal radioactivity at the same location of the enhanced signals in MR imaging. **(B)** ^18^F-S5 probe generated a significantly higher tumor-to-background ratio than the ^18^F-FDG probe (2.71 vs. 0.58, *p* = 0.006).

The PET imaging for ^18^F-S5 was conducted 60 min after injection of the radiotracer through the tail vein. ^18^F-S5 generated focal radioactivity at the site of meninges which is at the same location of enhanced signals generated by Gd-S5 in the previous MR imaging, whereas the brain parenchyma did not show significant radioactivity (Figure 4A). For the clinical commonly used PET radiotracer ^18^F-FDG, it generated diffuse enhanced radioactivity in the brain tissue due to the hypermetabolism of glucose (Figure 4A). The tumor-to-background ratio of the ^18^F-S5 probe was significantly higher than that of the ^18^F-FDG probe (2.71 vs. 0.58, *p* = 0.006; Figure 4B).

### HE Staining and Immunohistochemistry

**FIGURE 5:**
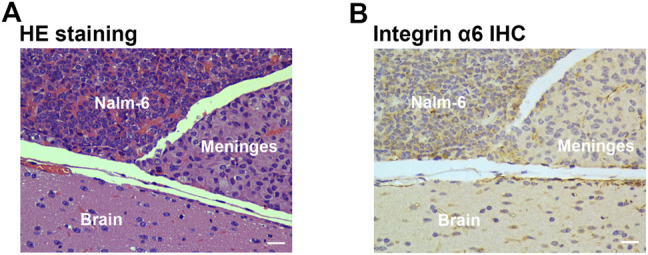
Pathological examination of tumor tissue. HE staining **(A)** identified layer-displayed leukemic cells in the superficial part of the brain parenchyma tissue, and immunohistochemical staining **(B)** confirmed the integrin α6 overexpression of the lesion (Scale bar, 40 μm).

At the end of the imaging experiments, mice were euthanized, and tumor tissues were removed for pathological examination. Pathological examination identified layer-displayed leukemic cells in the superficial part of the brain parenchyma tissue, and immunohistochemical staining confirmed the overexpression of integrin α6 within the lesion (Figure 5).

## Discussion

To date, the detection of CNS-L still remains a great challenge due to the lack of accurate diagnostic tools. CNS-L could occur at any time during the full course of leukemia, when the disease is apparently well-controlled even during relapse. Current diagnostic approaches including CNS symptoms and pathological detection of leukemic cells in the cerebrospinal fluid are generally hysteretic than the primary CNS involvement. Imaging detection of CNS-L lesions could be more distinct. MRI provides excellent soft tissue contrast and spatial resolution without ionizing radiation, which has been widely used due to its relatively low cost and easy accessibility (Mirzaei and Adeli, 2018). Meanwhile, PET imaging has been widely utilized in the diagnosis and management of cancer patients with relatively high sensitivity and quantitative-imaging ability (Shankar et al., 2020; Overcast et al., 2021). However, these imaging examinations have showed a limited ability in the imaging of CNS-L mainly due to the special histopathological characteristics of the disease.

Meningeal infiltration through the penetration of sinuses and small blood vessels is the earliest pathological process at the occurrence of CNS-L, followed by parenchymal infiltration, leukostasis, meningitis, intracranial edema, hemorrhage, and herniation (Yao et al., 2018; Halsey and Escherich, 2021). Distinctive from the other intracranial tumors, solid lesions hardly formed during the course of CNS-L, which caused a great challenge for conventional imaging methods (Pfeifer et al., 2003; Izraeli and Eckert, 2017). In addition, because of the physiological glucose hypermetabolism of a normal brain tissue, the most commonly used PET tracer ^18^F-FDG was less applicable to the intracranial lesions. Based on the premises, the development of novel molecular imaging probes with targeted ability could offer novel opportunities for the imaging of CNS-L.

Here, we focused on the potential tumor biomarker integrin α6, which was found to be overexpressed in a series of cancers (Huang et al., 2012; Brooks et al., 2016; Beaulieu, 2018). Physiologically, integrin α6 was mainly involved in cell proliferation, migration, survival, and differentiation by mediating cell-to-cell and cell-to-stroma adhesion. The expression and function of integrin α6 were abnormally altered in tumor cells, which promoted tumorigenesis, invasion, angiogenesis, metastasis, immune escape, and tolerance to radiotherapy and chemotherapy (Lathia et al., 2010; Beaulieu, 2019). In ALL, integrin α6 has been implicated to be crucial in the migration into CNS tissues and promote the survival of minimal residual disease after chemotherapy (Yao et al., 2018). Besides, silencing of integrin α6 could induce apoptosis and sensitize ALL cells to nilotinib or chemotherapy regimens, suggesting its important role in mediating chemoresistance (Yamakawa et al., 2012; Gang et al., 2020). As a membrane protein, integrin α6 also had a relatively long N-terminal extracellular domain that could be approached and bounded by targeted agents and which rendered it a potentially suitable target for molecular imaging and antitumor therapy.

In this study, the overexpression of integrin α6 was identified in numerous ALL cell lines including NALM6, REH, and CCRF-CEM cells, and the dominating membrane location of integrin α6 and the S5 peptide was confirmed by confocal microscopy. Then, the S5 peptide-based NIRF-, MRI-, and PET-targeted imaging probes were successfully constructed in four major domains: the S5 peptide domain for tumor targeting; Cy5, Gd, or ^18^F for the NIRF tracer, MRI contrast enhancement, or PET radiotracer; DOTA or NOTA as a chemical complexation ligand for linking Gd or ^18^F; and a PEG4 spacer to avoid the steric hindrance of the Cy5, Gd-DOTA, or ^18^F-NOTA monoamide to the S5 peptide. The synthesized agents Cy5-S5, Gd-S5, and ^18^F-S5 were hydrophilic and had good water solubility, suggesting its potential for further imaging development.

Our results illustrated that the gradually enhanced MRI signals were acquired at the site of meninges that were located between the skull bone and brain parenchyma of the mouse models, which was in accordance with the primary intracranial infiltrating location of CNS-L (Yao et al., 2018), reaching the peak at around 10 min, which was significantly highlighted compared to the surrounded brain tissues and easy to be recognized in visual, whereas the MRI images of the control group did not show significant enhancement signals. The quantitative signal analysis revealed that the S5 peptide-based probe generated, in approximately, signal enhancement 3 folds more than the control agent within the first 5 min post-injection, and the gap widened to nearly 4 folds at 10 min after injection. The signal enhancement gradually decreased thereafter and was still distinctive at 15 min post-injection. The PET imaging also showed regional radiation concentration at the same site of the MRI signal enhancement. Although the absolute SUV value was not high, its relative ratio to the brain parenchymal tissue reached near 3 folds, indicating considerable imaging ability of the S5-based PET probe.

Tumor-specific antibodies have been developed for targeted imaging; however, typically several days were needed to achieve the optimal imaging condition due to the long blood circulation duration of the antibodies. Targeted peptides have several advantages compared to the antibodies including the small size, sufficient capillary permeability, low immunogenicity, short biological half-time, rapid clearance from non-target tissues, ease of manufacture, and readily labeled with specific nuclides (Lee et al., 2010; Yao et al., 2016; Araste et al., 2018). In our study, Gd-S5 and ^18^F-S5 could be quickly excreted through the kidney due to its small molecular structure and good permeability, which enables the basis for imaging safety.

On the other hand, our study also has several limitations. First, the diagnostic efficacy of the molecular probes, compared to conventional cerebrospinal fluid examinations that are routinely conducted in clinics, was not investigated and could be further identified in larger animal models in which the cerebrospinal fluid is more accessible. Besides, the binding affinity of the S5 peptide to integrin α6 could be further enhanced through optimizing the peptide structure.

In conclusion, the study demonstrated that integrin α6 could serve as a feasible target for the molecular targeted imaging of CNS-L. The S5 peptide-based MRI and PET enhancement probes showed considerable ability in the imaging of CNS-L lesions in mouse models, indicating their potential for further development and clinical translation.

## Data Availability

The original contributions presented in the study are included in the article/Supplementary Material, further inquiries can be directed to the corresponding authors. The authenticity of this article has been validated by uploading the key raw data onto the Research Data Deposit public platform (www.researchdata.org.cn), with the approval RDD number as RDDB2022504768.
