# Supplementary figures and images for: Integrin α6-Targeted Molecular Imaging of Central Nervous System Leukemia in Mice
Source: Front Bioeng Biotechnol. 2022 Feb 23;10:812277. doi: 10.3389/fbioe.2022.812277 (PMC8905628; doi:10.3389/fbioe.2022.812277)

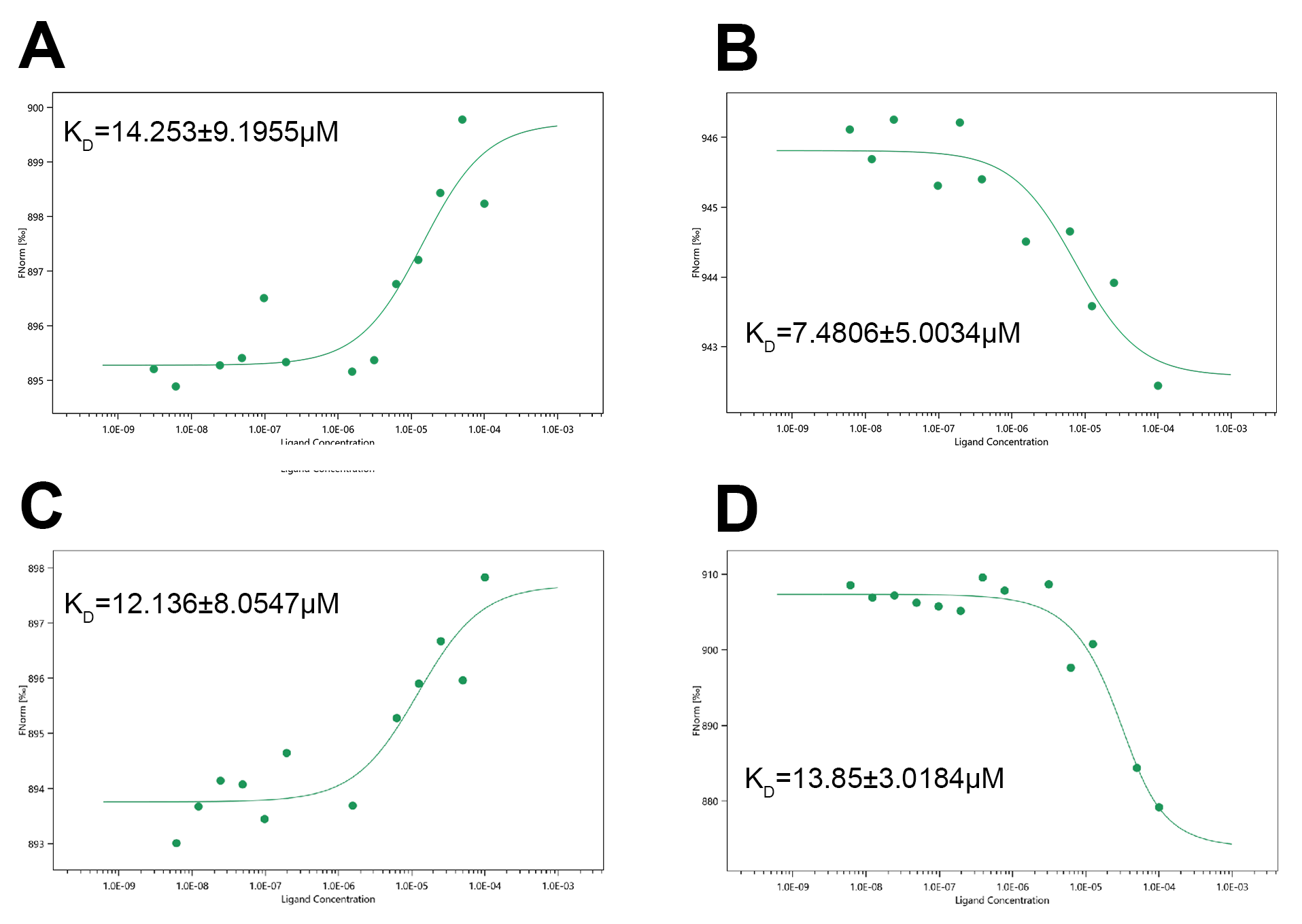

Supplement: Supplementary file 1 [file Image2.tif]

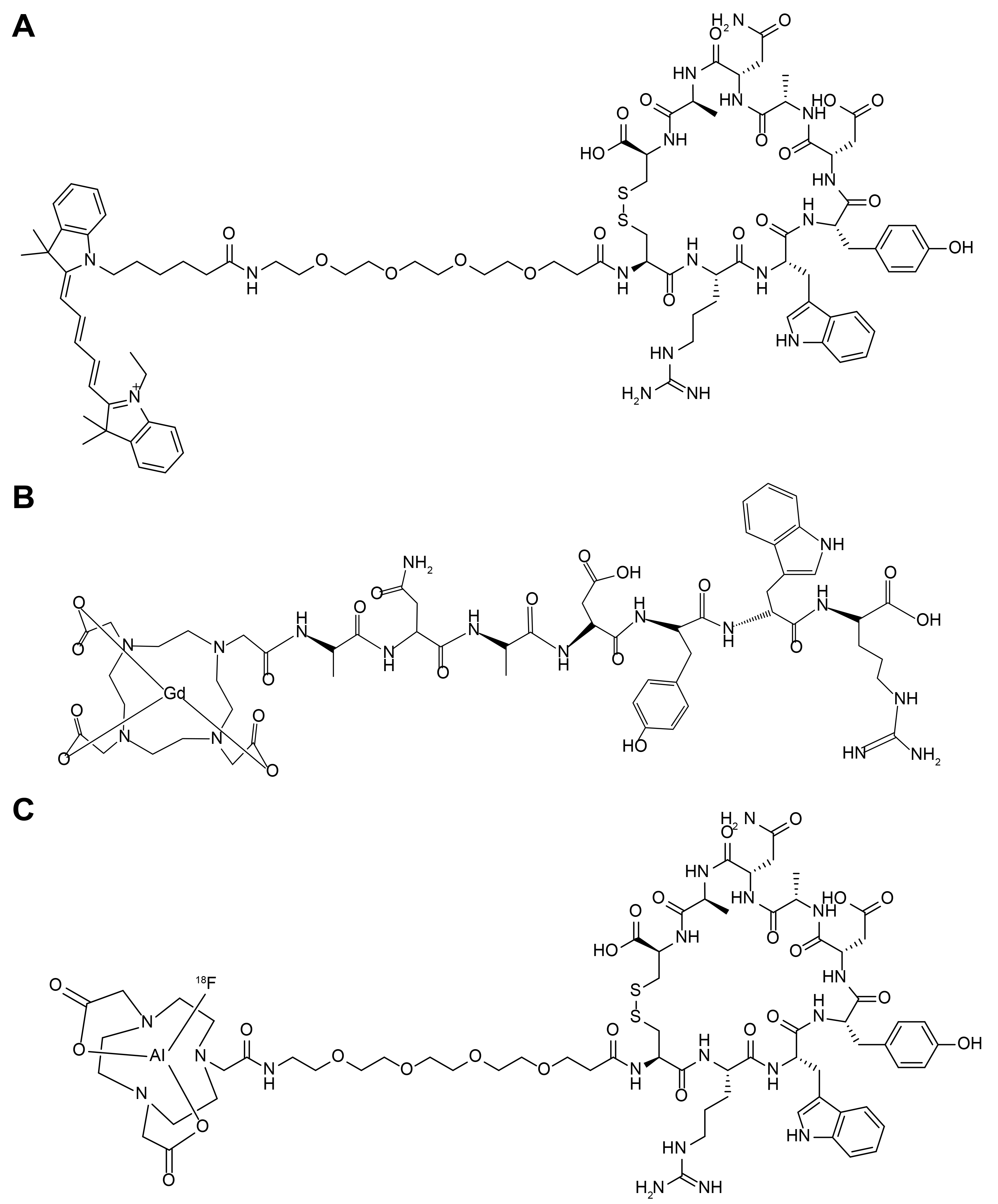

Supplement: Supplementary file 2 [file Image1.TIF]
