# Supplementary material for: Integrin α6-Targeted Molecular Imaging of Central Nervous System Leukemia in Mice
Source: Front Bioeng Biotechnol. 2022 Feb 23;10:812277. doi: 10.3389/fbioe.2022.812277 (PMC8905628; doi:10.3389/fbioe.2022.812277)
Supplement: Supplementary file 3 [file DataSheet1.docx]

**Supplementary file**

**Microscale thermophoresis**

The affinity of cyclic S5 peptide, straight S5 peptide, NOTA-S5 and Gd-S5 with Human ITGA6 & ITGB4 Heterodimer Protein (Manufactured by Sino Biological Catalog Number: CT069-H2508H) was determined by microscale thermophoresis (MST). Firstly, 0.05% PBST was prepared, 25ul of Tween 20 was added to 50ml of PBS and filtered through 0.22um filter paper. Then dilute the peptide: dissolve the above 4 peptides into 1mM storage solution concentration. Take 20 ul of peptide and mix with 80 ul of PBST and dilute to a working concentration of 200 nM. Next dilute the dye: Take 2 µL of RED-tris-NTA II dye (Manufactured by NanoTemper Cat# MO-L018 5 µM) and mix with 98 µl of PBST to obtain 100 µL of dye (100 nM). Finally, 100 ul of Human ITGA6 & ITGB4 Heterodimer Protein (400 nM) was mixed with 100 ul of dye (100 nM) and incubated at room temperature for 30 min, at which time the protein concentration was 200 nM. 10 ul of peptide (200 uM) was added to the PCR tube, and 16 tubes were diluted 1:1 with PBST. Then take 3 ul of protein/dye mixture in the PCR tube, add 3 ul of 4 different concentrations of peptide and mix well. The concentration of protein was 100 nM and the concentration of peptide in the first tube was 100 uM. Finally, the samples were aspirated with a capillary tube and assayed on the machine (Monolith NT.115 made in Germany) with the instrument set to 80 % LED/excitation power and medium MST power. Kd was calculated by Kd fitting mode using MO.Affinity Analysis.
